# Postpartum Body Mass Index Change Is Associated with Incident Dysglycemia in Women with a History of Gestational Diabetes Mellitus: A Prospective Cohort Study

**DOI:** 10.3390/jcm15072634

**Published:** 2026-03-30

**Authors:** Ryuto Tsushima, Asami Ito, Maika Oishi, Kana Ishihara, Kaori Iino, Kanji Tanaka, Yoshihito Yokoyama

**Affiliations:** 1 Department of Obstetrics and Gynecology, Graduate School of Medicine, Hirosaki University, Hirosaki 036-8563, Aomori, Japan; ancienregime729@gmail.com (R.T.); oishi120@hirosaki-u.ac.jp (M.O.); iino-ka@hirosaki-u.ac.jp (K.I.); yokoyama@hirosaki-u.ac.jp (Y.Y.); 2 Aomori Prefectural Central Hospital, Aomori City 030-8553, Aomori, Japan; 3 Odate Municipal General Hospital, Odate 017-8550, Akita, Japan; kana.smile.5917@gmail.com; 4 Hirosaki General Medical Center, Hirosaki 036-8562, Aomori, Japan; kanji@hirosaki-u.ac.jp

**Keywords:** gestational diabetes mellitus, postpartum weight change, impaired glucose tolerance, type 2 diabetes, dyslipidemia, cohort study

## Abstract

**Background/Objective:** Women with a history of gestational diabetes mellitus (GDM) are at increased risk of type 2 diabetes mellitus (T2DM), dysglycemia, and dyslipidemia. However, the role of postpartum weight change in long-term metabolic outcomes remains unclear. Here, we determined the long-term incidence of dysglycemia and dyslipidemia after GDM and evaluated whether postpartum changes in body mass index (BMI) independently predicted these outcomes. **Methods:** This single-center prospective cohort study included 205 Japanese women diagnosed with GDM. All participants underwent a 75 g oral glucose tolerance test at 6–12 weeks postpartum. The incidence of impaired fasting glucose (IFG), impaired glucose tolerance (IGT), T2DM, and dyslipidemia was evaluated over a median follow-up of 3.6 years. Cumulative incidence was estimated using the Kaplan–Meier method, and Cox proportional hazards models identified independent risk factors, particularly postpartum BMI change. **Results:** During follow-up, 42.4%, 6.3%, and 35.6% of women developed IFG or IGT (prediabetes), T2DM, and dyslipidemia, respectively. The estimated cumulative incidence rates at 6 years postpartum were 57.1% and 50% for IFG/IGT and dyslipidemia, respectively, whereas the 5-year incidence of T2DM was 10.3%. Postpartum BMI increase was independently associated with new-onset dysglycemia. No independent predictor of T2DM progression was identified. Dyslipidemia was independently associated with higher pre-pregnancy BMI and multiparity, whereas postpartum BMI change was not independently associated after multivariable adjustment. **Conclusions:** Postpartum BMI change was independently associated with dysglycemia in women with a history of GDM. These findings suggest that postpartum weight change may help identify women at higher risk of subsequent metabolic abnormalities, particularly dysglycemia, in this high-risk population, although causal relationships cannot be inferred from this observational study.

## 1. Introduction

Gestational diabetes mellitus (GDM) is defined as glucose intolerance first recognized during pregnancy, which typically resolves after delivery [1,2]. O’Sullivan reported that approximately 40% of women with a history of GDM develop type 2 diabetes mellitus (T2DM) within 20 years [3]. A history of GDM has major implications for long-term maternal health. Importantly, the time to progression varies across individuals, and both early and late postpartum conversion to T2DM after a GDM pregnancy have been reported [4]. Long-term follow-up studies indicate that women with a history of GDM are at high risk of developing T2DM after pregnancy [5,6]. Bellamy et al. estimated a relative risk (RR) of 7.4 (95% confidence interval [CI] 4.8–11.5) for T2DM in women with a history of GDM compared with women with normoglycemic pregnancies [5]. More recent meta-analyses suggest that the risk may approach 10-fold in some settings [6]. Without intervention, individuals with impaired glucose tolerance (IGT) have a substantially increased risk of progression to T2DM over time [7]. Women with obesity are at a particularly higher risk: a 20-year follow-up study in Finland reported that women with a history of GDM and normal weight had a 10.6-fold higher risk than controls, whereas the risk increased 47.2-fold among women with GDM and pre-pregnancy overweight or obesity [8].

Hyperglycemia and a history of GDM are associated with an increased risk of metabolic disorders. Meta-analyses indicate that women with a history of GDM have an approximately 2.5-fold higher risk of developing metabolic syndrome [9], and GDM is considered an important risk factor for subsequent metabolic disorders, including metabolic syndrome, which in turn increases the risk of cardiovascular diseases (CVDs) [10,11]. A 2019 meta-analysis of over 5 million women reported an approximately two-fold higher risk of major CVD events (RR 1.98) and women who did not progress to T2DM showed a 56% higher risk (RR 1.56) in those with a history of GDM [12]. These findings underscore that GDM is a marker of long-term health risks beyond the index pregnancy.

Accordingly, clinical guidelines emphasize the importance of long-term monitoring and preventive interventions in women with a history of GDM. The American Diabetes Association (ADA) recommends a 75 g oral glucose tolerance test (OGTT) at 4–12 weeks postpartum, followed by ongoing glycemic screening every 1–3 years [13]. The National Institute for Health and Care Excellence recommends a fasting plasma glucose (FPG) test at 6–13 weeks postpartum to exclude diabetes; if diabetes is not diagnosed, annual HbA1c testing is recommended thereafter [14]. In Australia, women are advised to undergo a 75 g OGTT at 6–12 weeks postpartum, be informed of their increased risk of developing T2DM, and undergo regular diabetes screening every 1–3 years [15,16,17]. The World Health Organization guideline recommends a 75 g OGTT at 6–8 weeks postpartum, followed by annual diabetes screening thereafter [18]. Japanese guidelines similarly recommend a 75 g OGTT at 6–12 weeks postpartum and continued periodic assessment of glucose tolerance in women with prior GDM [19]. In high-risk women (e.g., those with a history of GDM with prediabetes or elevated glycated hemoglobin [HbA1c]), prophylactic metformin may be considered [7,13,20]. In East Asian women, Kawasaki et al. reported that the concurrence of pre-pregnancy obesity (body mass index [BMI] ≥ 25 kg/m^2^), IGT within 12 weeks postpartum, and HbA1c ≥ 5.7% was associated with an odds ratio of approximately 51 for subsequent T2DM, highlighting the value of early postpartum glycemic assessment for long-term risk stratification [21].

However, the effect of postpartum weight change on long-term metabolic outcomes remains insufficiently characterized. Although it has long been speculated that weight gain after a GDM pregnancy may elevate the risk of future diabetes and metabolic syndrome, relatively few studies have quantified this effect in women with GDM. Recent systematic reviews have highlighted postpartum weight change as a potentially modifiable factor influencing long-term metabolic health after pregnancy, as sustained weight gain may contribute to the progression of glucose intolerance and other cardiometabolic disturbances [22].

In a Chinese cohort, Liu et al. demonstrated that postpartum weight gain substantially increased the risk of subsequent diabetes in women with previous GDM, whereas Kaiser et al. reported a high prevalence of metabolic syndrome in this population [10,23]. To date, no study has specifically examined how the degree of BMI change (ΔBMI) after delivery influences long-term prognosis in women with GDM. Addressing this gap is important because, unlike fixed factors, such as age or genetics, body weight is modifiable through lifestyle interventions. Demonstrating the relationship between postpartum weight change and outcomes may inform strategies to improve long-term health in this population.

Therefore, we conducted a prospective cohort study among women with a history of GDM to investigate long-term metabolic outcomes. The primary objective of this study was to determine the 5–6-year cumulative incidence of postpartum impaired fasting glucose (IFG) or IGT (prediabetes), T2DM, and dyslipidemia in this high-risk group. The secondary objective was to identify independent risk factors for these outcomes, with a particular focus on changes in BMI after delivery. We hypothesized that an increase in BMI after delivery represents an independent and modifiable determinant of long-term metabolic deterioration, including progression of glucose intolerance and the development of dyslipidemia, regardless of baseline metabolic risk factors. Specifically, we assumed that women who experience sustained postpartum BMI gain have a significantly higher risk of developing abnormalities in glucose and lipid metabolism compared with those who maintain or reduce their body weight.

Thus, this study was designed not only to quantify the cumulative incidence of metabolic disorders after GDM, but also to determine whether longitudinal changes in BMI independently predict future metabolic outcomes.

## 2. Materials and Methods

### 2.1. Study Design and Population

The GDM Postpartum in the Aomori Prefecture (GDM-PPAP) study is a single-center prospective cohort study designed to evaluate long-term metabolic outcomes in women with a history of GDM. The study was conducted at Hirosaki University Hospital (Aomori, Japan), a tertiary academic referral center serving northern Japan. A dedicated postpartum follow-up clinic for women with GDM was established in January 2014 to provide structured metabolic surveillance after delivery.

Participants were consecutively recruited between January 2014 and December 2021 (Data collection began in 2016), and follow-up continued until December 2024. The study was conducted in accordance with the Declaration of Helsinki and was approved by the Ethics Committee of Hirosaki University Graduate School of Medicine (Approval No. 2016-158; approved on 1 November 2016). Written informed consent was obtained from all participants prior to inclusion, and all data were anonymized before analysis to protect participant confidentiality. The study design and reporting adhered to the Strengthening the Reporting of Observational Studies in Epidemiology (STROBE) guidelines.

### 2.2. Study Population

Eligibility criteria were as follows: (1) diagnosis of GDM during pregnancy according to the criteria of the Japanese Association of Diabetes and Pregnancy (equivalent to the International Association of Diabetes and Pregnancy Study Groups (IADPSG) thresholds based on a 75 g OGTT) [19], (2) delivery of a singleton pregnancy at Hirosaki University Hospital, and (3) at least one year of postpartum follow-up.

Exclusion criteria included pre-existing type 1 or type 2 diabetes, chronic kidney disease, endocrine disorders affecting glucose metabolism, autoimmune disease, severe systemic illness, or pregnancy complications requiring specialized metabolic management. Women diagnosed with overt diabetes at the 6–12 weeks postpartum evaluation were excluded from analyses of incident outcomes. These criteria ensured that the study population consisted of women with GDM without other major metabolic or systemic disorders that could substantially influence metabolic outcomes during pregnancy or postpartum follow-up. Previous history of GDM was defined as a diagnosis of GDM in a pregnancy prior to the index pregnancy.

**Figure 1:**
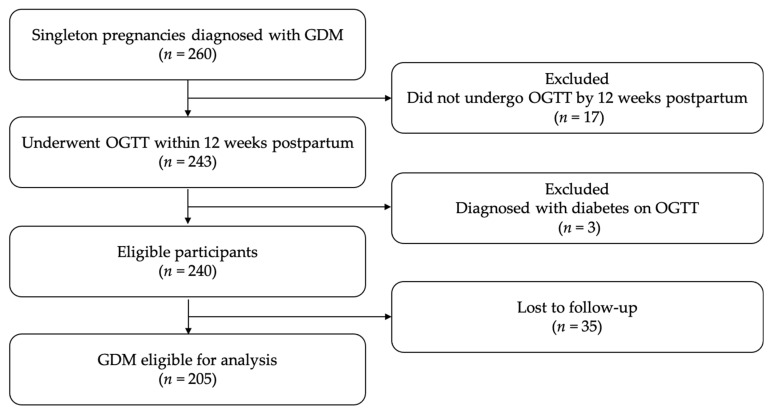
Flow diagram of participant selection in the GDM-PPAP study. Among 260 women diagnosed with GDM, 243 underwent a postpartum OGTT within 12 weeks. After excluding three women diagnosed with overt diabetes and 35 lost to follow-up, 205 women were included in the final analysis.

The participant selection process is summarized in Figure 1. During the study period, 260 women with singleton pregnancies were diagnosed with GDM at our institution. Of these, 243 underwent a 75 g OGTT within 12 weeks postpartum. Three women were excluded because overt diabetes was diagnosed at the postpartum OGTT. Among the remaining eligible participants (*n* = 240), 35 were lost to follow-up during the observation period. Consequently, 205 women with a history of GDM were included in the final analysis. The selection and follow-up of participants are illustrated in a flow diagram (Figure 1).

### 2.3. Postpartum Follow-Up Protocol

At 6–12 weeks postpartum, all participants underwent a comprehensive metabolic evaluation, including a 75 g OGTT. IGT was defined as a 2 h plasma glucose level of 140–199 mg/dL [24], while IFG was defined as a fasting plasma glucose level of 110–125 mg/dL [25]. T2DM was defined as meeting any ADA diagnostic criterion (FPG ≥ 126 mg/dL, 2 h OGTT glucose ≥ 200 mg/dL, or HbA1c ≥ 6.5%) or a physician diagnosis with initiation of diabetes medications [25]. Dyslipidemia was defined according to the Japan Atherosclerosis Society guidelines [26]: low-density lipoprotein cholesterol ≥ 140 mg/dL, high-density lipoprotein cholesterol < 40 mg/dL, triglycerides ≥ 150 mg/dL, or initiation of lipid-lowering therapy. Glucose-related outcomes were defined using established diagnostic criteria from international guidelines (WHO criteria for IFG/IGT and ADA criteria for T2DM), whereas dyslipidemia was defined according to the Japan Atherosclerosis Society guidelines. These diagnostic thresholds were based on internationally recognized clinical guidelines to ensure standardized outcome definitions and comparability with previous studies. Based on the 6–12 weeks results, participants entered a structured follow-up schedule. Women with normal postpartum OGTT results (no IFG, IGT, or T2DM) were advised to undergo annual follow-up visits for repeat OGTT and metabolic screening. Women with abnormal 6–12 weeks OGTT results (IFG, IGT, or overt diabetes) were followed more frequently (approximately every 6 months) according to the clinical protocol. Women diagnosed with overt diabetes at 6–12 weeks postpartum were referred to the endocrinology department for management and excluded from analyses of “new-onset” outcomes. Incident metabolic outcomes during follow-up were identified based on laboratory measurements obtained at follow-up visits and physician diagnoses documented in the medical records. Participants received individualized counseling on diet, physical activity, and weight management at each visit, consistent with standard postpartum care for GDM at the institution. During follow-up, 33 of the 205 participants (16.1%) experienced a subsequent pregnancy. If a participant became pregnant again during the follow-up period, postpartum BMI change was calculated using the pre-pregnancy BMI immediately before the subsequent pregnancy and the BMI at the last follow-up visit, in order to reduce distortion by gestational weight gain. For time-to-event analyses, follow-up time was not paused or interrupted at the onset of a subsequent pregnancy; rather, it was calculated continuously from the index delivery to the first documented outcome or the last follow-up visit, and a subsequent pregnancy was not treated as a separate censoring event.

### 2.4. Study Measurements

Pre-pregnancy BMI was calculated using height and preconception or first trimester weight. Body weight was measured at each postpartum follow-up visit using calibrated digital scales, and BMI was calculated as weight in kilograms divided by height in meters squared. “Postpartum BMI change” was defined as the difference between BMI at the one-month postpartum visit and BMI at the last follow-up visit. In women who experienced an intervening pregnancy during follow-up, BMI change was calculated from the pre-pregnancy BMI before the new pregnancy to the BMI at the last follow-up visit. Annualized BMI change (ΔBMI per year) was also calculated by dividing the total BMI change by the individual follow-up duration; however, because follow-up duration varied only modestly, total BMI change over the entire follow-up period was used as the primary exposure variable in the analyses. For descriptive and survival analyses, participants were further categorized into tertiles of postpartum BMI change: (a) “weight loss or minimal gain” (lowest tertile), (b) “moderate weight gain” (middle tertile), and (c) “high weight gain” (highest tertile).

Blood samples for glucose and lipid measurements were obtained after an overnight fast of at least 8 h and analyzed in the hospital central laboratory using standardized enzymatic methods.

### 2.5. Outcome Definitions

The study outcomes were incident IFG/IGT, incident T2DM, and incident dyslipidemia during follow-up. The primary outcomes were incident IFG/IGT and incident T2DM, whereas the secondary outcome was incident dyslipidemia.

Time-to-event was calculated continuously from the date of the index delivery to the first documented occurrence of the respective outcome, with participants without an event censored at the date of their last follow-up visit. Thus, follow-up time was not paused or interrupted at the onset of a subsequent pregnancy, and a subsequent pregnancy during follow-up was not treated as a separate censoring event in the present analysis.

### 2.6. Statistical Analysis

Normality of continuous variables was assessed using the Shapiro–Wilk test. Normally distributed variables are presented as mean ± standard deviation and were compared using Student’s *t*-test, whereas non-normally distributed variables are presented as median (interquartile range) and were compared using the Mann–Whitney U test. Categorical variables are expressed as counts and percentages and were compared using the chi-square test or Fisher’s exact test, as appropriate.

Time-to-event analyses were performed using the Kaplan–Meier method to estimate the cumulative incidence of metabolic outcomes. Differences between groups were assessed using the log-rank test. Kaplan–Meier curves were also constructed according to postpartum BMI categories (<25 vs. ≥25 kg/m^2^ at the 1-month postpartum visit) and tertiles of postpartum BMI change.

Cox proportional hazards regression models were used to estimate hazard ratios (HRs) and 95% CIs for predictors of metabolic outcomes. Candidate covariates included maternal age at delivery, pre-pregnancy BMI, previous history of GDM, parity, family history of diabetes, insulin treatment during the index GDM pregnancy, OGTT results during pregnancy (0, 1, and 2 h), OGTT results at 6–12 weeks postpartum, and postpartum BMI change. Variables considered for the multivariable models were selected based on both statistical significance in univariable analyses and clinical relevance according to previous literature. Key clinically relevant covariates, such as maternal age, pre-pregnancy BMI, and postpartum glucose measurements, were also considered during model building to account for potential confounding. For the analysis of incident IFG/IGT, all candidate variables were first examined in univariable Cox proportional hazards models, and variables with *p* < 0.05 in the univariable analyses were subsequently entered into the multivariable model. Similarly, for the analysis of incident T2DM, all candidate variables were first examined in univariable Cox proportional hazards models, and variables with *p* < 0.05 were subsequently entered into the multivariable model. Likewise, for the analysis of incident dyslipidemia, all candidate variables were first examined in univariable Cox proportional hazards models, and variables with *p* < 0.05 were subsequently entered into the multivariable model. To assess potential multicollinearity among variables entered into the multivariable model, pairwise Spearman’s correlation coefficients and variance inflation factors (VIFs) were evaluated. Because no meaningful multicollinearity was detected, the selected variables were entered simultaneously into the multivariable Cox model. The proportional hazards assumption was evaluated using Schoenfeld residuals.

Missing data were handled using complete-case analysis. For the IFG/IGT, T2DM, and dyslipidemia analyses, postpartum OGTT values recorded as 0 were treated as missing values. Because the multivariable analyses for incident IFG/IGT, incident T2DM, and incident dyslipidemia were based on complete-case data, the number of analyzed participants varied according to variable availability. Sensitivity analyses related to short follow-up duration were not included in the primary analyses.

To minimize potential bias, consecutive eligible patients were recruited during the study period. Outcome ascertainment was standardized within a structured clinical follow-up protocol, and laboratory measurements were performed using validated methods in a certified central laboratory. All statistical tests were two-sided, and *p* values < 0.05 were considered statistically significant.

This study included all eligible women during the recruitment period; therefore, no formal a priori sample size calculation was performed. However, assuming an expected cumulative incidence of approximately 30% for abnormal glucose metabolism and an anticipated hazard ratio of 2.0 associated with higher postpartum BMI gain, a sample size of approximately 200 participants would provide an estimated statistical power of 80% at a two-sided alpha level of 0.05.

All analyses were performed using SPSS version 25 for Windows (IBM Inc., Armonk, NY, USA).

## 3. Results

### 3.1. Cohort Characteristics

A total of 205 women with a history of GDM were included in the analysis. Baseline characteristics of the study population are summarized in Table 1. The mean age at delivery was 35.4 ± 5.0 years. The mean pre-pregnancy BMI was 23.7 ± 5.2 kg/m^2^, and the mean gestational weight gain was 5.9 ± 5.3 kg. Nearly half of the participants were primiparous (49.0%), and 50.2% had a family history of diabetes. The mean gestational age at GDM diagnosis was 18.4 ± 7.7 weeks. Based on the diagnostic 75 g OGTT, 63.9% of participants had one-point abnormalities, 29.2% had two-point abnormalities, and 5.3% met all three abnormality criteria. During pregnancy, 25.9% required insulin therapy in addition to dietary management. Regarding delivery outcomes, 61.5% underwent vaginal delivery and 38.5% delivered by cesarean section. The mean gestational age at delivery was 38.3 ± 2.4 weeks, and 8.8% experienced preterm delivery (<37 weeks). The mean birth weight was 2943.7 ± 516.4 g and mean umbilical artery blood pH was 7.31 ± 0.07. During follow-up, 33 participants (16.1%) experienced a subsequent pregnancy.

### 3.2. Incidence of Postpartum Outcomes

**Figure 2:**
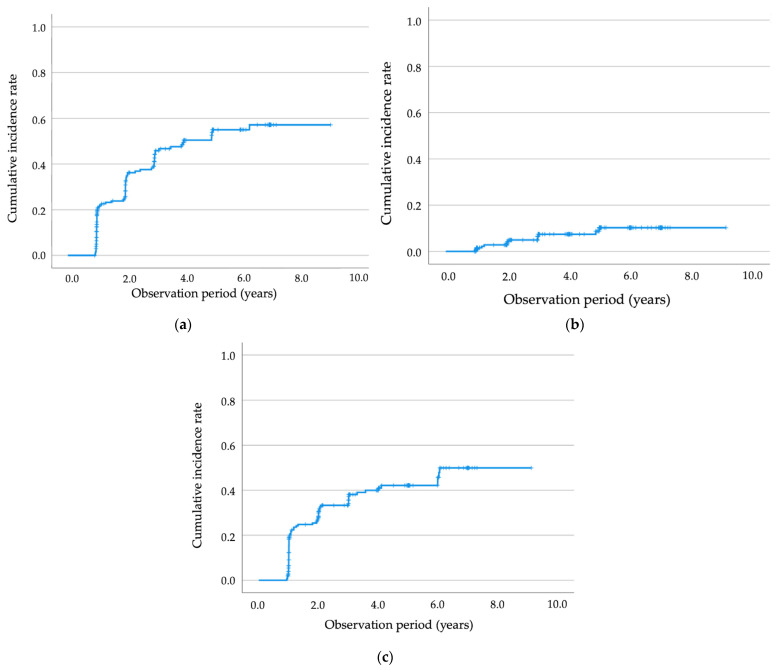
Kaplan–Meier curves showing the cumulative incidence of metabolic outcomes during the 10-year observation period among women with a history of GDM. (**a**) IFG or IGT; (**b**) T2DM; (**c**) dyslipidemia. Censoring is indicated by tick marks on the curves. Abbreviations: GDM, gestational diabetes mellitus; IFG, impaired fasting glucose; IGT, impaired glucose tolerance; T2DM, type 2 diabetes mellitus.

Over a median follow-up of 3.6 years, a substantial proportion of participants developed metabolic abnormalities. At the last follow-up visit, 87 women (42.4%) had developed IFG or IGT, 13 (6.3%) had developed T2DM, and 73 (35.6%) had developed dyslipidemia. In the time-to-event analyses, follow-up was calculated continuously from the index delivery to the first documented outcome or the last follow-up visit, and a subsequent pregnancy was not treated as a separate censoring event. Figure 2 illustrates the cumulative incidence of IFG/IGT, T2DM, and dyslipidemia over time. By 6 years postpartum, the Kaplan–Meier–estimated cumulative incidence of IFG/IGT reached 57.1%, indicating that more than half of participants experienced abnormal glucose metabolism within this period. The cumulative incidence of dyslipidemia was approximately 50% at 6 years postpartum. In contrast, progression to T2DM was less frequent in the early postpartum period; by 5 years postpartum, approximately 10.3% of participants had developed T2DM.

### 3.3. Predictors of Postpartum IFG/IGT

**Figure 3:**
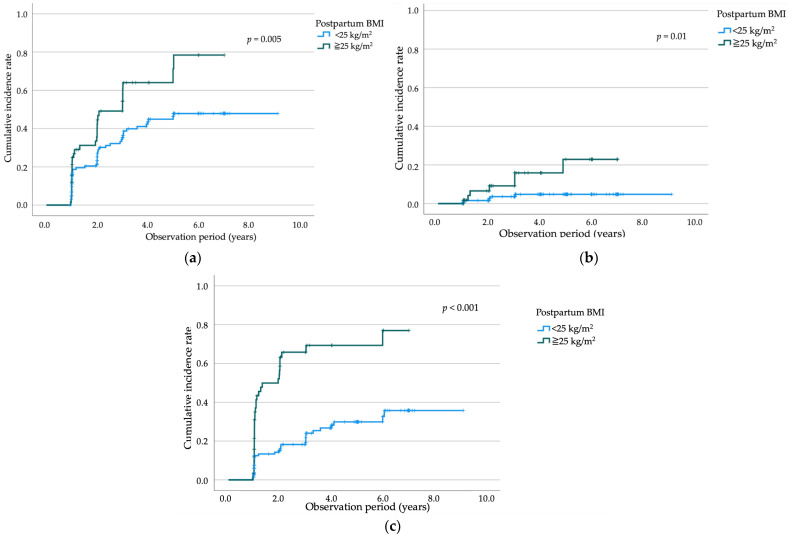
Kaplan–Meier curves showing the cumulative incidence of metabolic outcomes during the 10-year observation period among women with a history of GDM, stratified by postpartum BMI (<25 kg/m^2^ vs. ≥25 kg/m^2^). (**a**) Impaired glucose tolerance (IFG/IGT), with significantly higher incidence in women with postpartum BMI ≥ 25 kg/m^2^ (log-rank test, *p* = 0.005); (**b**) T2DM, with significantly higher incidence in women with postpartum BMI ≥ 25 kg/m^2^ (log-rank test, *p* = 0.01); (**c**) dyslipidemia, with a significantly higher incidence in women with postpartum BMI ≥ 25 kg/m^2^ (log-rank *p* < 0.001). Abbreviations: GDM, gestational diabetes mellitus; IFG, impaired fasting glucose; IGT, impaired glucose tolerance; T2DM, type 2 diabetes mellitus; BMI, body mass index; *p*, probability value; log-rank test, Mantel–Cox test.

Kaplan–Meier analyses demonstrated that postpartum BMI was significantly associated with the risk of glucose intolerance during follow-up. As illustrated in Figure 3a, participants with a postpartum BMI ≥ 25 kg/m^2^ had a higher cumulative incidence of IFG/IGT than those with a postpartum BMI < 25 kg/m^2^ (log-rank *p* = 0.005). The survival curves diverged early and continued to separate over time, indicating a sustained increase in dysglycemia risk among women with elevated postpartum BMI.

**Figure 4:**
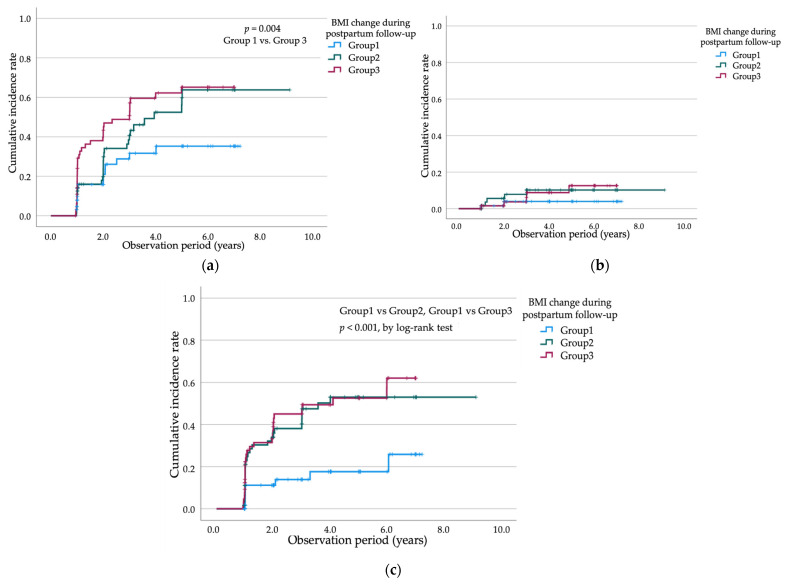
Kaplan–Meier curves showing the cumulative incidence of metabolic outcomes during the 10-year observation period among women with a history of GDM, stratified by postpartum BMI change (tertiles). (**a**) IFG or IGT, with a significantly higher cumulative incidence in Group 3 (highest tertile of BMI change) than in Group 1 (lowest tertile) (log-rank test, *p* = 0.004); (**b**) T2DM, with no significant differences observed among the three groups; (**c**) dyslipidemia, with significantly higher cumulative incidence in Group 2 and Group 3 than in Group 1 (log-rank *p* < 0.001). Abbreviations: GDM, gestational diabetes mellitus; IFG, impaired fasting glucose; IGT, impaired glucose tolerance; T2DM, type 2 diabetes mellitus; BMI, body mass index; *p*, probability value; log-rank test, Mantel–Cox test.

Postpartum BMI change was associated with the development of dysglycemia. As shown in Figure 4a, the cumulative incidence of IFG/IGT was stratified by tertiles of postpartum BMI change, defined as the difference between BMI at the 1-month postpartum visit and BMI at the last follow-up visit. In Kaplan–Meier analyses, participants in the highest tertile of BMI gain exhibited the greatest risk of developing IFG/IGT. By 6 years postpartum, >60% of women in the high BMI gain group developed IFG or IGT, compared with approximately 45% in the moderate gain group and 30% in the low/no gain group, with a significant difference among groups (log-rank *p* = 0.004).

To comprehensively re-evaluate predictors of incident IFG/IGT, all variables included in Table 2 were first examined in univariable Cox proportional hazards analyses. In the univariable analyses, multiparity (HR 0.61, 95% CI 0.40–0.94; *p* = 0.024), pregnancy OGTT 2 h glucose (HR 1.01, 95% CI 1.00–1.01; *p* = 0.003), postpartum OGTT 1 h glucose (HR 1.01, 95% CI 1.01–1.02; *p* < 0.001), postpartum OGTT 2 h glucose (HR 1.01, 95% CI 1.01–1.02; *p* < 0.001), and change in BMI during follow-up (HR 1.13, 95% CI 1.03–1.25; *p* = 0.012) were significantly associated with incident IFG/IGT, whereas maternal age at delivery, pre-pregnancy BMI, previous history of GDM, family history of diabetes, insulin therapy, pregnancy OGTT fasting glucose, pregnancy OGTT 1 h glucose, and postpartum OGTT fasting glucose were not significantly associated with incident IFG/IGT.

Variables with *p* < 0.05 in the univariable analyses were subsequently entered into the multivariable Cox model. No meaningful multicollinearity was detected among these variables (maximum Spearman’s ρ = 0.54; all VIFs < 1.33). In the complete-case multivariable model (*n* = 187; 80 events), higher pregnancy OGTT 2 h glucose (HR 1.01, 95% CI 1.00–1.01; *p* = 0.005), higher postpartum OGTT 1 h glucose (HR 1.01, 95% CI 1.00–1.02; *p* = 0.011), and greater BMI change during follow-up (HR 1.15, 95% CI 1.04–1.27; *p* = 0.006) remained independently associated with incident IFG/IGT. In contrast, postpartum OGTT 2 h glucose was no longer statistically significant after adjustment (HR 1.01, 95% CI 1.00–1.01; *p* = 0.226), and multiparity showed borderline significance (HR 0.64, 95% CI 0.41–1.00; *p* = 0.051) (Table 2).

### 3.4. Predictors of Postpartum T2DM

Kaplan–Meier analysis demonstrated that postpartum BMI was significantly associated with T2DM development. As shown in Figure 3b, participants with a postpartum BMI ≥ 25 kg/m^2^ had a significantly higher cumulative incidence of T2DM than those with a BMI < 25 kg/m^2^ (log-rank *p* = 0.010). In contrast, when participants were categorized into tertiles based on postpartum BMI change (ΔBMI), no significant difference in the cumulative incidence of T2DM was observed among the three groups (Figure 4b). To comprehensively re-evaluate predictors of incident T2DM, all variables included in Table 3 were first examined in univariable Cox proportional hazards analyses. In the univariable analyses, previous history of GDM (HR 5.15, 95% CI 1.63–16.26; *p* = 0.005), insulin therapy (HR 3.64, 95% CI 1.22–10.88; *p* = 0.021), pregnancy OGTT 1 h glucose (HR 1.02, 95% CI 1.00–1.04; *p* = 0.048), postpartum OGTT 1 h glucose (HR 1.03, 95% CI 1.01–1.05; *p* < 0.001), and postpartum OGTT 2 h glucose (HR 1.02, 95% CI 1.01–1.04; *p* = 0.002) were significantly associated with incident T2DM, whereas maternal age at delivery, pre-pregnancy BMI, multiparity, family history of diabetes, pregnancy OGTT fasting glucose, pregnancy OGTT 2 h glucose, postpartum OGTT fasting glucose, and BMI change during follow-up were not significantly associated with incident T2DM.

Variables with *p* < 0.05 in the univariable analyses were subsequently entered into the multivariable Cox model. No meaningful multicollinearity was detected among these variables. In the complete-case multivariable model (*n* = 195; 12 events), previous history of GDM remained borderline associated with incident T2DM (HR 3.79, 95% CI 1.00–14.38; *p* = 0.050), whereas insulin therapy, pregnancy OGTT 1 h glucose, postpartum OGTT 1 h glucose, and postpartum OGTT 2 h glucose were not independently associated with incident T2DM after adjustment (Table 3).

### 3.5. Predictors of Postpartum Dyslipidemia

A similar pattern was observed for dyslipidemia. As shown in Figure 3c, participants with a postpartum BMI ≥ 25 kg/m^2^ had a significantly higher cumulative incidence of dyslipidemia than those with a postpartum BMI < 25 kg/m^2^ (log-rank *p* < 0.001). The cumulative incidence curves progressively diverged over the follow-up period, indicating a strong association between elevated postpartum BMI and subsequent lipid abnormalities.

Figure 4c illustrates the cumulative incidence of dyslipidemia stratified by tertiles of postpartum BMI change, defined as the difference between BMI at the 1-month postpartum visit and BMI at the last follow-up visit. Participants with greater postpartum BMI gain showed a markedly higher cumulative incidence of dyslipidemia. The cumulative incidence curves demonstrated a clear separation among groups, with both the middle (Group 2) and highest (Group 3) tertiles of BMI change exhibiting significantly higher risk than the lowest tertile (Group 1), as indicated by a significant log-rank test (*p* < 0.001).

To comprehensively evaluate predictors of incident dyslipidemia, all variables listed in Table 4 were first examined in univariable Cox proportional hazards analyses. In the univariable analyses, higher pre-pregnancy BMI (HR 1.11, 95% CI 1.07–1.15; *p* < 0.001), multiparity (HR 0.52, 95% CI 0.32–0.83; *p* = 0.006), and greater BMI change during follow-up (HR 1.18, 95% CI 1.06–1.30; *p* = 0.002) were significantly associated with incident dyslipidemia, whereas maternal age at delivery, previous history of GDM, insulin therapy, family history of diabetes, pregnancy OGTT fasting, 1 h, and 2 h glucose, postpartum OGTT fasting glucose, postpartum OGTT 1 h glucose, and postpartum OGTT 2 h glucose were not significantly associated with incident dyslipidemia.

Variables with *p* < 0.05 in the univariable analyses were subsequently entered into the multivariable Cox model. No meaningful multicollinearity was detected among these variables (maximum Spearman’s ρ = 0.43; all VIFs < 1.20). In the complete-case multivariable model (*n* = 193; 68 events), higher pre-pregnancy BMI remained independently associated with incident dyslipidemia (HR 1.11, 95% CI 1.07–1.15; *p* < 0.001), whereas multiparity remained inversely associated with dyslipidemia risk (HR 0.53, 95% CI 0.32–0.86; *p* = 0.011). In contrast, BMI change during follow-up was no longer statistically significant after adjustment (HR 1.05, 95% CI 0.94–1.17; *p* = 0.418) (Table 4).

## 4. Discussion

One finding of this study was that greater postpartum BMI change was independently associated with incident IFG/IGT during follow-up. Among women with a history of GDM, even relatively small increases in BMI after pregnancy were associated with a significantly higher risk of subsequent dysglycemia. Specifically, each 1-unit increase in BMI was associated with an approximately 15% higher risk of developing IFG or IGT. In the multivariable analysis, higher pregnancy OGTT 2 h glucose, higher postpartum OGTT 1 h glucose, and greater BMI change during follow-up remained independently associated with incident IFG/IGT. These findings suggest that postpartum weight change may be clinically relevant to long-term metabolic outcomes in women with a history of GDM. The findings are consistent with the Kaplan–Meier analyses, which showed that both elevated postpartum BMI (≥25 kg/m^2^) and greater postpartum BMI increases (upper tertile of ΔBMI) were associated with a substantially higher cumulative incidence of IFG/IGT over time. Together, these findings are consistent with the possibility that post-pregnancy weight management may be relevant to the long-term trajectory of glucose metabolism in women at high risk.

In the present cohort, progression to frank T2DM in the early postpartum years was relatively uncommon, whereas a substantial proportion of participants developed prediabetes or dyslipidemia within 6 years. These findings indicate that health risks following GDM are not limited to immediate conversion to diabetes. Instead, many women experience a gradual deterioration in metabolic health, manifested as impaired glucose regulation or features of metabolic syndrome during the first few years after delivery. Prediabetes is clinically important, as it is associated with an increased risk of CVD and frequently progresses to diabetes if left unaddressed [27,28]. By 6 years postpartum, nearly 57% of the cohort had developed IFG and/or IGT. Kaplan–Meier analyses showed that a postpartum BMI ≥ 25 kg/m^2^ was significantly associated with a higher T2DM incidence, whereas tertiles of ΔBMI did not show significant differences. In the multivariable analysis for T2DM, no clear independent predictor was identified after adjustment. These findings should be interpreted cautiously because the complete-case multivariable analysis included only 12 T2DM events, which limited statistical power and may have resulted in imprecise estimates. Accordingly, the multivariable findings for incident T2DM should be considered exploratory. These findings suggest the importance of early and sustained surveillance of glucose and lipid metabolism in women with a history of GDM, beginning soon after childbirth.

The present results support and extend the findings of previous studies. Long-term follow-up studies have consistently demonstrated that women with a history of GDM are at high risk of developing T2DM after delivery [5,6]. In a large meta-analysis, Bellamy et al. estimated a relative risk of 7.4 (95% CI 4.8–11.5) for T2DM in women with a history of GDM compared with those with normoglycemic pregnancies [5]. More recent meta-analyses have suggested that this risk may approach 10-fold in certain populations [6]. Moreover, in the absence of preventive interventions, women with a history of GDM have a substantially increased long-term risk of progression to T2DM, with some cohorts reporting a high cumulative incidence within the first decade postpartum [6]. In contrast, the 5-year incidence of T2DM in the present study was approximately 10%. This discrepancy may be attributed to several factors. First, the median follow-up duration (3.6 years) was shorter than 10 years; therefore, additional cases of diabetes may have developed beyond the observation period. Second, ethnic differences may have contributed. The study population consisted exclusively of Japanese women, who generally have lower BMI than their Western counterparts. In addition, East Asian populations exhibit distinct relationships between insulin sensitivity and β-cell response compared with other ethnic groups, which may modify the risk trajectory from GDM to T2DM [29,30]. Third, all participants received proactive postpartum follow-up and lifestyle guidance, which may have contributed to improved metabolic monitoring and may have influenced the progression to diabetes. Large randomized controlled trials, such as the Diabetes Prevention Program (DPP), have demonstrated that targeted interventions in high-risk individuals can substantially delay or prevent the development of T2DM. In the DPP, intensive lifestyle modification reduced the incidence of diabetes by 58%, and metformin therapy decreased the incidence by 31% compared with placebo [7]. Furthermore, among women with impaired glucose tolerance and a history of GDM, postpartum improvements in lifestyle behaviors and metformin use have been reported to delay or prevent the onset of T2DM [20,31]. However, these intervention effects were not evaluated in the present study.

Although this study was not an interventional trial, some women may have adopted healthier lifestyle behaviors postpartum, such as dietary improvement or increased physical activity. These factors may partly explain the relatively low 5-year incidence of T2DM observed in this cohort. However, because these lifestyle factors were not systematically measured, their contribution cannot be directly assessed in the present study. Likewise, the potential influence of pharmacological prevention could not be evaluated. Therefore, any interpretation regarding the roles of diet, physical activity, or pharmacological intervention in the present cohort should be considered hypothesis-generating rather than directly supported by our data. Nevertheless, previous studies have shown that lifestyle modification and pharmacological therapy can reduce the risk of diabetes in women with a history of GDM [7,20,31].

An important finding of this study was that postpartum BMI change remained independently associated with incident IFG/IGT, even after adjustment for glycemic indicators during pregnancy and the early postpartum period. Previous studies of GDM outcomes have often evaluated weight change in categorical terms (e.g., comparing weight retention with weight loss). In contrast, BMI change was analyzed as a continuous variable, allowing quantification of risk increase per unit gain. A clear dose–response relationship was observed; for example, a +3 kg/m^2^ increase in BMI (approximately an +8 kg increase in body weight) was associated with an approximately 50% higher risk of developing prediabetes. These findings suggest that even modest postpartum weight gain is associated with an increased risk of dysglycemia, whereas weight maintenance or reduction after delivery may be associated with a lower risk of glucose intolerance and diabetes progression. From a clinical perspective, these findings suggest that postpartum weight management may be relevant to long-term glucose risk stratification in women with a history of GDM.

Regarding dyslipidemia, pre-pregnancy BMI, parity, and 2 h glucose levels at the early postpartum OGTT were identified as independent predictors. These findings suggest that baseline adiposity and early postpartum metabolic status may play an important role in determining long-term lipid abnormalities in women with a history of GDM. In contrast to dysglycemia, postpartum BMI change itself was not an independent predictor of dyslipidemia after adjustment for confounders. This difference may indicate that lipid metabolism may be more strongly influenced by baseline metabolic status than by short-term weight change after pregnancy.

These findings are consistent with evidence from high-risk populations showing that weight reduction may reduce the risk of developing T2DM [7,32]. However, because the present study was observational, causal effects of weight change cannot be inferred. The results suggest that avoiding postpartum weight gain may be associated with a lower risk of dysglycemia in women with a history of GDM.

These observations are logical from a pathophysiological perspective. Pregnancy is a state of progressive insulin resistance. After delivery, failure to lose gestational weight or additional weight gain may allow insulin resistance to persist or worsen. This chronic insulin-resistant state places continued stress on an already vulnerable pancreatic β-cell system, particularly in women with a history of GDM, in whom both β-cell dysfunction and insulin resistance are associated with future diabetes risk [33]. Over time, this process can lead to β-cell failure and the development of IFG or IGT. In contrast, women who successfully return to pre-pregnancy weight (or below) may experience improved insulin sensitivity, thereby reducing the metabolic load on β-cells and maintaining normal glucose tolerance for longer periods. These findings align with this mechanistic understanding and represent the first clinical quantification of its effect in a GDM follow-up setting. In addition, the independent predictive roles of 2 h glucose levels during pregnancy OGTT and 1 h glucose levels during the early postpartum OGTT, as identified in the Cox analysis, further underscore the prognostic importance of glycemic severity during and shortly after pregnancy. Collectively, the results provide real-world evidence that postpartum weight change may be associated with metabolic outcomes in women with a history of GDM.

Interestingly, postpartum BMI change was not an independent predictor of dyslipidemia, whereas pre-pregnancy BMI remained independently associated with incident dyslipidemia. This finding suggests that the risk of postpartum dyslipidemia may be more strongly related to long-term obesity status (baseline adiposity) than to short-term weight fluctuations. Women with chronic overweight or obesity (high pre-pregnancy BMI) are prone to dyslipidemia regardless of modest weight changes, likely due to long-standing insulin resistance and altered lipid metabolism. This interpretation is consistent with the Kaplan–Meier findings showing a higher incidence of dyslipidemia among women with postpartum BMI ≥ 25 kg/m^2^ and those in the upper ΔBMI tertile, although only pre-pregnancy BMI and multiparity remained independently associated with incident dyslipidemia in the multivariable Cox analysis. Postpartum OGTT indices were not independently associated with dyslipidemia after adjustment.

From a clinical perspective, this study highlights three main points. First, regular glucose screening is important in women with a history of GDM. A substantial proportion of IFG/IGT cases occurred within 1–3 years after delivery. Without periodic testing, these cases may remain undetected until progression to diabetes. Regular postpartum screening, as recommended by the ADA and JDS guidelines [13,19], enabled the early identification of metabolic deterioration, allowing timely interventions. Second, the importance of weight management and lifestyle interventions in this population should be considered. Postpartum weight gain was associated with prediabetes risk, whereas avoidance of weight gain was associated with a lower risk. These findings may support consideration of dietary counseling, physical activity promotion, and weight monitoring as components of postpartum care for women with GDM history. However, because lifestyle behaviors and pharmacological prevention were not systematically measured in this study, the effects of these approaches cannot be directly inferred from our data. The relevance of such interventions in this context should therefore be interpreted cautiously and warrants further investigation in future interventional studies. Evidence from prevention trials confirms that even moderate lifestyle modifications can substantially reduce progression to T2DM in high-risk individuals [7,20], but these interventional findings should not be interpreted as being directly demonstrated by the present study. Third, a comprehensive approach to postpartum follow-up is required. Women with a history of GDM are at an elevated risk not only for diabetes but also for related metabolic and cardiovascular conditions; therefore, follow-up care should address broad health aspects. This includes monitoring for dyslipidemia, hypertension, and glycemic status, with early intervention when abnormalities are detected. Coordinated care among obstetrics, primary care, and endocrinology is ideal to ensure appropriate long-term surveillance and preventive management after a GDM pregnancy.

This study has several limitations that should be considered when interpreting the results. First, information on breastfeeding practices was not systematically collected. Because lactation may influence postpartum weight change and metabolic outcomes, residual confounding cannot be excluded. Second, lifestyle factors such as diet, physical activity, and pharmacological prevention were not systematically measured in this study. Therefore, although these factors may potentially influence long-term metabolic outcomes, their contribution could not be directly evaluated, and the related interpretations should be considered exploratory. Third, during follow-up, 33 of the 205 participants (16.1%) experienced a subsequent pregnancy. Because a subsequent pregnancy is not a random event and may be associated with both postpartum BMI trajectories and the risk of subsequent metabolic outcomes, informative censoring and exposure contamination cannot be completely excluded. In addition, the interconception interval was not explicitly analyzed in the present study. Therefore, the observed associations should be interpreted as reflecting long-term metabolic risk after an index GDM pregnancy under real-world reproductive trajectories, rather than an isolated non-pregnant postpartum course. Fourth, the number of incident T2DM events was limited, which reduced statistical power for the multivariable T2DM analysis and may have contributed to imprecise estimates. Finally, this study was conducted at a single tertiary center and included only Japanese women, which may limit the generalizability of the findings to other populations with different ethnic or healthcare backgrounds. Future multicenter studies with larger cohorts and more detailed assessments of lifestyle factors are warranted to confirm these findings.

## 5. Conclusions

Postpartum BMI change was independently associated with incident dysglycemia in women with a history of GDM. Higher pregnancy OGTT 2 h glucose and higher postpartum OGTT 1 h glucose were also independently associated with incident IFG/IGT. In contrast, postpartum BMI change was not independently associated with incident dyslipidemia after adjustment, whereas higher pre-pregnancy BMI and multiparity remained independently associated with dyslipidemia. For incident T2DM, no clear independent predictor was identified in the multivariable analysis, and the limited number of T2DM events warrants cautious interpretation.

These findings suggest that postpartum BMI change may help identify women with a history of GDM who are at higher risk of subsequent metabolic abnormalities, particularly dysglycemia. However, because this was an observational study, causal inferences cannot be made. Further prospective and interventional studies are needed to determine whether postpartum weight management and other targeted strategies can improve long-term metabolic outcomes in women with a history of GDM.

## Figures and Tables

**Table 1:** Patient characteristics (*n* = 205).

Characteristic	Value
Age at delivery (years)	35.4 ± 5.0
Pre-pregnancy BMI (kg/m^2^)	23.7 ± 5.2
Gestational weight gain (kg)	5.9 ± 5.3
Primiparous	100 (49.0%)
Family history of diabetes	103 (50.2%)
Gestational week at GDM diagnosis	18.4 ± 7.7
OGTT	
One-point positive	131 (63.9%)
Two-point positive	60 (29.2%)
Three-point positive	11 (5.3%)
Insulin therapy	53 (25.9%)
Mode of delivery	
Vaginal delivery	126 (61.5%)
Cesarean section	79 (38.5%)
Gestational age at delivery (weeks)	38.3 ± 2.4
Preterm delivery (<37 weeks)	18 (8.8%)
Birth weight (g)	2943.7 ± 516.4
Umbilical artery blood pH	7.31 ± 0.07

Values are presented as mean ± SD or *n* (%). Abbreviations: BMI, body mass index; GDM, gestational diabetes mellitus; OGTT, oral glucose tolerance test.

**Table 2:** Univariable and multivariable Cox proportional hazards analyses for incident IFG/IGT.

Variable	Univariable HR (95% CI)	Univariable *p*-Value	Multivariable HR (95% CI)	Multivariable *p*-Value
Maternal age at delivery (years)	1.00 (0.96–1.05)	0.894	—	—
Pre-pregnancy BMI (kg/m^2^)	1.03 (1.00–1.07)	0.066	—	—
Previous history of GDM	0.83 (0.42–1.66)	0.606	—	—
Multiparous	0.61 (0.40–0.94)	0.024	0.64 (0.41–1.00)	0.051
Family history of diabetes	1.45 (0.95–2.22)	0.085	—	—
Insulin therapy	1.47 (0.93–2.33)	0.096	—	—
Pregnancy OGTT				
Fasting glucose	1.00 (0.98–1.02)	0.648	—	—
1 h glucose	1.01 (1.00–1.01)	0.119	—	—
2 h glucose	1.01 (1.00–1.01)	0.003	1.01 (1.00–1.01)	0.005
Postpartum OGTT				
Fasting glucose	1.00 (0.98–1.03)	0.802	—	—
1 h glucose	1.01 (1.01–1.02)	<0.001	1.01 (1.00–1.02)	0.011
2 h glucose	1.01 (1.01–1.02)	<0.001	1.01 (1.00–1.01)	0.226
Change in BMI during follow-up (per +1 kg/m^2^)	1.13 (1.03–1.25)	0.012	1.15 (1.04–1.27)	0.006

Values are presented as HRs with 95% CIs from Cox proportional hazards models for incident IFG/IGT during follow-up. Univariable analyses were first performed for all variables included in Table 2, and variables with *p* < 0.05 were entered into the multivariable model. Postpartum OGTT values recorded as 0 were treated as missing. Multicollinearity among variables entered into the multivariable model was assessed using Spearman’s correlation coefficients and variance inflation factors (VIFs), and no meaningful multicollinearity was detected. The multivariable model was based on complete-case analysis (*n* = 187; 80 events). Abbreviations: IFG, impaired fasting glucose; IGT, impaired glucose tolerance; GDM, gestational diabetes mellitus; OGTT, oral glucose tolerance test; BMI, body mass index; HR, hazard ratio; CI, confidence interval.

**Table 3:** Univariable and multivariable Cox proportional hazards analyses for incident T2DM.

Variable	Univariable HR (95% CI)	Univariable *p*-Value	Multivariable HR (95% CI)	Multivariable *p*-Value
Maternal age at delivery (years)	1.02 (0.91–1.15)	0.731	—	—
Pre-pregnancy BMI (kg/m^2^)	1.06 (0.97–1.16)	0.196	—	—
Multiparous	0.90 (0.30–2.75)	0.86	—	—
Previous history of GDM	5.15 (1.63–16.26)	0.005	3.79 (1.00–14.38)	0.050
Insulin therapy	3.64 (1.22–10.88)	0.021	1.08 (0.26–4.41)	0.919
Family history of diabetes	1.54 (0.52–4.60)	0.439	—	—
Pregnancy OGTT	—	—	—	—
Fasting glucose	1.03 (0.99–1.07)	0.202	—	—
1 h glucose	1.02 (1.00–1.04)	0.048	1.00 (0.98–1.02)	0.757
2 h glucose	1.01 (0.99–1.02)	0.392	—	—
Postpartum OGTT	—	—	—	—
Fasting glucose	1.03 (0.97–1.09)	0.318	—	—
1 h glucose	1.03 (1.01–1.05)	<0.001	1.02 (1.00–1.04)	0.089
2 h glucose	1.02 (1.01–1.04)	0.002	1.01 (0.99–1.03)	0.307
Change in BMI during follow-up (per +1 kg/m^2^)	1.13 (0.89–1.44)	0.319	—	—

Values are presented as HRs with 95% CIs from Cox proportional hazards models for incident T2DM during follow-up. Univariable analyses were first performed for all variables included in Table 3, and variables with *p* < 0.05 were entered into the multivariable model. Postpartum OGTT values recorded as 0 were treated as missing. Multicollinearity among variables entered into the multivariable model was assessed using Spearman’s correlation coefficients and variance inflation factors (VIFs), and no meaningful multicollinearity was detected. The multivariable model was based on complete-case analysis (*n* = 195; 12 events). Abbreviations: T2DM, type 2 diabetes mellitus; GDM, gestational diabetes mellitus; OGTT, oral glucose tolerance test; BMI, body mass index; HR, hazard ratio; CI, confidence interval.

**Table 4:** Univariable and multivariable Cox proportional hazards analyses for incident dyslipidemia.

Variable	Univariable HR (95% CI)	Univariable *p*-Value	Multivariable HR (95% CI)	Multivariable *p*-Value
Maternal age at delivery (years)	1.04 (0.99–1.09)	0.106	—	—
Pre-pregnancy BMI (kg/m^2^)	1.11 (1.07–1.15)	<0.001	1.11 (1.07–1.15)	<0.001
Multiparous	0.52 (0.32–0.83)	0.006	0.53 (0.32–0.86)	0.011
Previous history of GDM	0.98 (0.49–1.97)	0.956	—	—
Insulin therapy	1.39 (0.85–2.29)	0.191	—	—
Family history of diabetes	1.46 (0.91–2.32)	0.113	—	—
Pregnancy OGTT	—	—	—	—
Fasting glucose	1.02 (1.00–1.03)	0.106	—	—
1 h glucose	1.00 (1.00–1.01)	0.487	—	—
2 h glucose	1.00 (1.00–1.01)	0.244	—	—
Postpartum OGTT	—	—	—	—
Fasting glucose	1.01 (0.99–1.04)	0.282	—	—
1 h glucose	1.00 (1.00–1.01)	0.603	—	—
2 h glucose	1.01 (1.00–1.02)	0.052	—	—
Change in BMI during follow-up (per +1 kg/m^2^)	1.18 (1.06–1.30)	0.002	1.05 (0.94–1.17)	0.418

Values are presented as HRs with 95% CIs from Cox proportional hazards models for incident dyslipidemia during follow-up. Univariable analyses were first performed for all variables included in Table 4, and variables with *p* < 0.05 were entered into the multivariable model. Postpartum OGTT values recorded as 0 were treated as missing. Multicollinearity among variables entered into the multivariable model was assessed using Spearman’s correlation coefficients and variance inflation factors (VIFs), and no meaningful multicollinearity was detected. The multivariable model was based on complete-case analysis (*n* = 193; 68 events). Abbreviations: GDM, gestational diabetes mellitus; OGTT, oral glucose tolerance test; BMI, body mass index; HR, hazard ratio; CI, confidence interval.

## Data Availability

The datasets used and/or analyzed during the current study are available from the corresponding author upon reasonable request.

## Abbreviations

The following abbreviations are used in this manuscript:

ADA	American Diabetes Association
AIC	Akaike information criterion
BMI	Body mass index
CVD	Cardiovascular disease
DPP	Diabetes prevention program
FPG	Fasting plasma glucose
GDM	Gestational diabetes mellitus
HbA1c	Glycated hemoglobin
HR	Hazard ratio
IFG	Impaired fasting glucose
IGT	Impaired glucose tolerance
JDS	Japanese Diabetes Society
NICE	National Institute for Health and Care Excellence
OGTT	Oral glucose tolerance test
T2DM	Type 2 diabetes mellitus
